# Long-term outcomes of laparoscopic versus open distal gastrectomy for patients with advanced gastric cancer in North China: a multicenter randomized controlled trial

**DOI:** 10.1007/s00464-024-10952-2

**Published:** 2024-07-09

**Authors:** Jiadi Xing, Jun Cai, Xiaohui Wang, Nengwei Zhang, Dali An, Fei Li, Ming Cui, Lei Niu, Chongchong Gao, Qing Fan, Shulin Ren, Zhongtao Zhang, Xiangqian Su, SWEET trial group

**Affiliations:** 1 Key Laboratory of Carcinogenesis and Translational Research (Ministry of Education), Department of Gastrointestinal Surgery IV, Peking University Cancer Hospital & Institute, Beijing, China; 2 Department of General Surgery, Beijing Friendship Hospital, Capital Medical University, Beijing, China; 3 Department of General Surgery, Beijing Xuanwu Hospital, Capital Medical University, Beijing, China; 4 Department of General Surgery, School of Clinical Medicine, Peking University Ninth, Beijing Shijitan Hospital, Capital Medical University, Beijing, China; 5 Department of General Surgery, Beijing Tongren Hospital, Capital Medical University, Beijing, China; 6 State Key Laboratory of Holistic Integrative Management of Gastrointestinal Cancers, Department of Gastrointestinal Surgery IV, Beijing Key Laboratory of Carcinogenesis and Translational Research, Peking University Cancer Hospital & Institute, Beijing, China

**Keywords:** Advanced gastric cancer, Distal gastrectomy, Laparoscopy, Disease-free survival, Body mass index

## Abstract

**Background:**

Laparoscopic distal gastrectomy (LDG) has become a common procedure for treating advanced gastric cancer (AGC) in China. However, there is uncertainty regarding its oncological outcomes compared to open distal gastrectomy (ODG). This study aims to compare the 3-year disease-free survival (DFS) rates among patients who underwent surgery for AGC in northern China.

**Methods:**

A multicenter, non-inferiority, open-label, parallel, randomized clinical trial was conducted to evaluate patients with AGC who were eligible for distal gastrectomy at five tertiary hospitals in North China. In this trial, patients were randomly assigned preoperatively to receive either LDG or ODG in a 1:1 allocation ratio. The primary endpoint was postoperative morbidity and mortality within 30 days and the secondary endpoint was the 3-year DFS rate. This trial has been registered at ClinicalTrials.gov (Identifier: NCT02464215).

**Results:**

A total of 446 patients were randomly allocated to LDG (*n = *223) or ODG group (*n = *223) between March 2014 and August 2017. After screening, a total of 214 patients underwent the open surgical approach, while 216 patients underwent laparoscopic surgery. The 3-year DFS rate was 85.9% for the LDG group and 84.72% for the ODG group, with no significant statistical difference (Hazard ratio 1.12; 95% CI 0.68–1.84, *P* = 0.65). Body mass index (BMI) < 25 kg/m^2^, advanced pathologic T4, and pathologic N2-3 category were confirmed as independent risk factors for DFS in the Cox regression.

**Conclusions:**

In comparison to ODG, LDG with D2 lymphadenectomy yielded similar outcomes in terms of 3-year DFS rates among patients diagnosed with AGC.

**Supplementary Information:**

The online version contains supplementary material available at 10.1007/s00464-024-10952-2.

## Introduction

Gastric cancer (GC) stands as the fifth most prevalent malignant tumor and the fourth leading cause of cancer-related mortality globally, as indicated by the GLOBOCAN 2020 data [1]. Despite the widespread adoption of gastroscopic examinations, over 80% of gastric cancer diagnoses in China manifest as advanced gastric cancer (AGC) [2]. Numerous high-quality clinical studies have affirmed the safety and viability of laparoscopic distal gastrectomy (LDG) in comparison to open distal gastrectomy (ODG) for AGC on a global scale [3, 4]. While laparoscopic techniques have demonstrated associations with reduced blood loss, decreased postoperative pain, and expedited recovery [3–5], controversies persist regarding the oncological prognosis of laparoscopy. This is primarily attributed to the potential of pneumoperitoneum to facilitate tumor cell propagation and escalate local recurrence [6, 7]. Furthermore, the oncological prognosis of LDG for AGC remains inadequately established due to insufficient comprehensive evidence derived from randomized clinical trials (RCTs). Two rigorous RCTs conducted in Korea (KLASS-02) and China (CLASS-01) have consecutively validated the absence of a substantial disparity in 3-year disease-free survival (DFS) rates between LDG and ODG for patients with AGC [8, 9]. Furthermore, another multi-center RCT in Netherlands (NCT02248519) is presently in the follow-up phase [10].

Hence, we hypothesized that the 3-year DFS rate in patients with AGC undergoing LDG is comparable to that of patients undergoing ODG. Previously, we had reported that LDG was a safe and feasible option for patients with AGC, when compared with ODG in short-term surgical outcomes [5]. Herein, this was the secondary study that reported on the long-term outcomes of laparoscopic versus open distal gastrectomy for patients with AGC in northern China.

## Materials and methods

### Study design

This multicenter, non-inferiority, open-label, parallel, randomized clinical trial involved nine surgeons from five tertiary hospitals in northern China. The study protocol received approval from the medical ethics committees of all participating hospitals. Additionally, the trial is registered at ClinicalTrial.gov (NCT02464215).

### Patients

The inclusion criteria for this study were as follows: (1) Patients aged over 18 years diagnosed with primary gastric adenocarcinoma via gastroscopy; (2) Tumor located in the lower or middle third of the stomach, with patients treated using either LDG or ODG with D2 lymph node dissection; (3) Preoperative clinical stage based on the American Joint Committee on Cancer (AJCC) 8th edition TNM guideline, ranging from Ib to IIIc (T2-4aN0-3M0); (4) American Society of Anesthesiology (ASA) Score I to III; (5) Patients who signed an informed consent form before surgery.

Exclusion criteria were: (1) History of upper abdominal surgery (excluding laparoscopic cholecystectomy); (2) Underwent endoscopic submucosal dissection or mucosal resection before surgery; (3) Presence of an enlarged lymph node with a diameter larger than 3 cm based on preoperative imaging; (4) History of chemotherapy, immunotherapy, or radiotherapy before surgery.

### Information of surgeons

All surgeons who met the following criteria were selected from the SWEET group: (1) had independently performed a minimum of 60 ODG and 60 LDG with D2 lymphadenectomy; (2) had conducted at least 80 gastrostomies annually; and (3) were confirmed as qualified surgeons by the SWEET group through evaluation of their comprehensive surgical videos. Ultimately, 13 *surgeons* from 5 institutions participated in this trial.

### Surgical quality control

With the surgical approaches, both procedures of LDG and ODG group were identical. The location of trocars was not limited while the total number should be less than five in LDG group. The extent of lymphadenectomy adhered principles of Japanese gastric cancer treatment guidelines. Reconstruction was performed in accordance with the surgeon’s preference and experience, either Billroth-I, Billroth-II, or Roux-en-Y gastrojejunostomy was performed during in surgery. In LDG group, only one mini-laparotomy incision less than 10 cm was acceptable. Moreover, ten photos were uploaded for each participant. Among them, five pictures were taken for lymph node dissection fields, four for the lesion and resection margins of specimens, and one for the abdominal incision.

### Randomization and masking

Randomization and masking procedures were overseen by the contract research organization (CRO, Beijing High-land Med-Tech Development, Beijing, China) throughout the trial. Participants, including both patients and surgeons, were not blinded to the intervention. Eligible patients were allocated randomly to either the ODG or LDG groups upon signing the informed consent form. Therefore, the blinding methods are not applicable to this study.

### Procedure and endpoint

Both the ODG and LDG groups underwent standard D2 lymphadenectomy, following the Japanese guidelines. The intricate surgical technique has been documented in our prior investigation [5]. The primary outcome assessed in this study was postoperative morbidity and mortality within 30 days and the secondary outcome was the 3-year DFS rates, defined as the duration from randomization to the occurrence of tumor progression, including local recurrence or metastasis.

### Adjuvant chemotherapy and follow-up

The decision regarding adjuvant chemotherapy was meticulously evaluated based on the tumor's pathological stage and the preferences of patients. Oxaliplatin-based chemotherapy regimens such as XELOX, FOLFOX, and SOX were recommended for patients with T3–4 tumors or any positive lymph nodes. Adjuvant therapy for all AGC patients is performed within 6 months following surgery in this study.

All participating patients were followed up for more than 36 months following surgery. Follow-up protocol was every three months for the first two years and then every six months for the next three years. Follow-up included (1) physical examination and blood test for serum tumor markers including carcinoembryonic antigen (CEA), cancer antigen 19–9, and cancer antigen 12–5; (2) imaging with abdominopelvic and chest computed tomographic scans; (3) annual gastrointestinal endoscopy. Magnetic resonance imaging, positron emission tomography, or laparoscopic exploration were used for final diagnosis in the event of suspicious results indicating local recurrence or metastasis. The last follow-up day was October 16, 2020.

### Statistical analysis

Statistical analyses were conducted using SPSS version 19.0 (IBM Corp., Armonk, NY, USA) and R version 3.6.2 (R Foundation). Continuous data were expressed as mean ± standard deviation and analyzed using Student’s t-test or Mann–Whitney U test. Categorical variables were assessed using the Chi-square test and Fisher’s exact test, and results were presented as frequency (%). The 3-year DFS rates were calculated using the Kaplan–Meier method. Univariate and multivariate analyses were performed using Cox regression models. Subgroup analysis was conducted for DFS stratified by pathological stage (I, II, III, IV) and lymph node status (N0 vs. N +) using the log-rank test. A *P*-value < 0.05 was considered statistically significant.

## Results

**Fig. 1:**
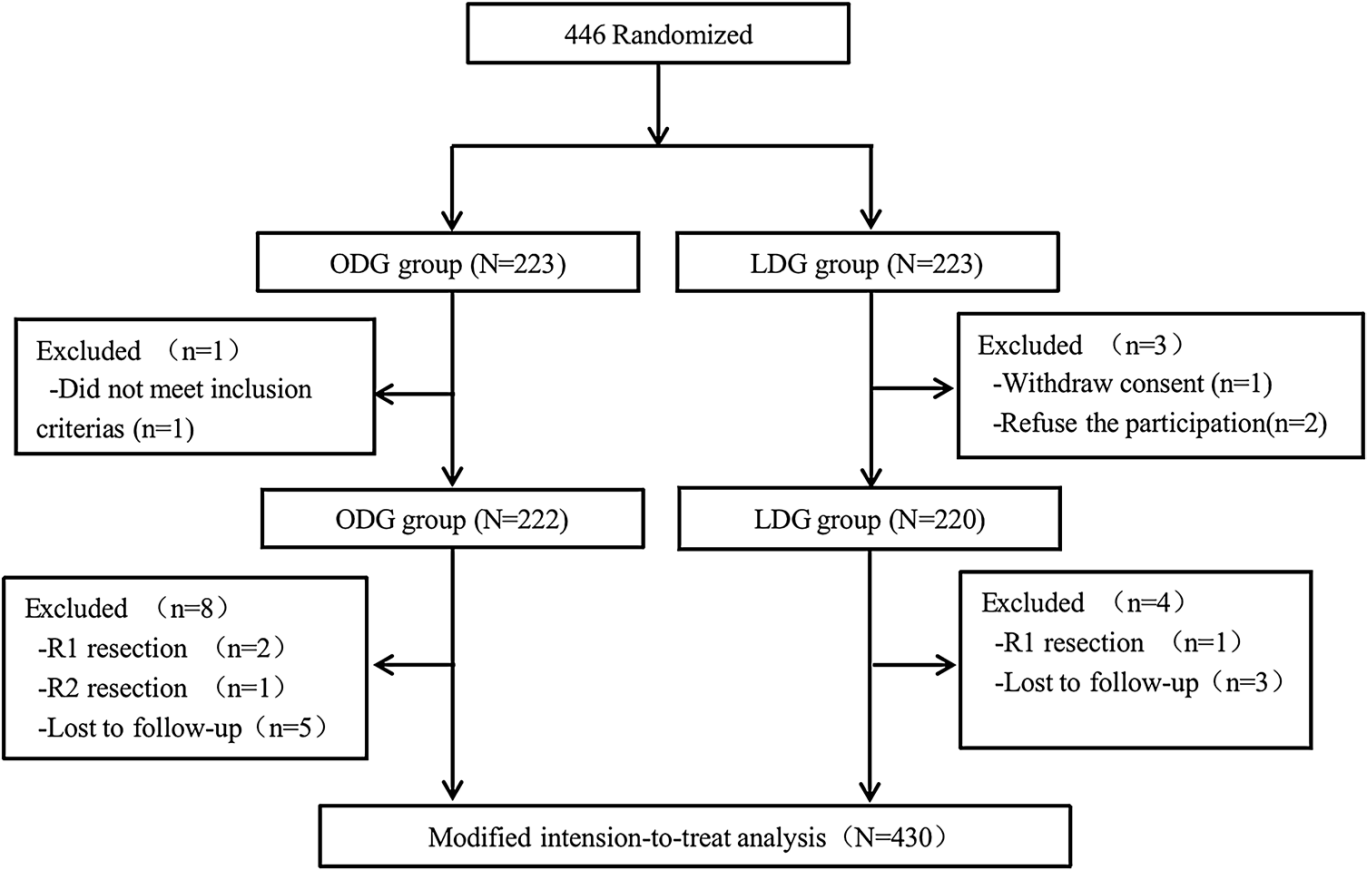
Flow of patient enrollment and randomization

A total of 446 patients diagnosed with AGC between March 2014 and August 2017 were recruited from five tertiary hospitals in North China. Among them, 214 patients were randomly assigned to the ODG group, while 216 patients were allocated to the LDG group for final analysis. Four patients were excluded post-randomization due to inappropriate reasons, as outlined in our previously published study [5]. Additionally, twelve patients (laparoscopy, *n = *8; Open, *n = *4) who underwent R1 or R2 resection and were subsequently lost to follow-up were excluded from the study. The flowchart illustrating the trial process is presented in Fig. 1.

### Clinicopathologic characteristics

The baseline clinical characteristics of the included patients are presented in Table 1. In the ODG group, there were 76 male patients, while in the LDG group, there were 85 male patients (*P* = 0.411). The mean age was 59 years in the ODG group and 60 years in the LDG group (*P* = 0.577), with mean BMIs of 23.26 kg/m^2^ and 23.46 kg/m^2^, respectively (*P* = 0.479). The two groups showed no significant differences in terms of comorbidities, ASA score, and Clinical TNM stage. Postoperative pathological data are also summarized in Table 2. Despite most patients initially diagnosed with clinical stage II or higher, 137 patients (34.1% in the ODG group and 29.6% in the LDG group) were confirmed to be at pathological stage I. The rates of patients receiving adjuvant chemotherapy were similar in both groups (laparoscopy, 47.7%; open surgery, 47.7%, *P* = 0.996). Additionally, there were no significant differences between the two groups in terms of retrieved lymph nodes and chemotherapy regimens.

**Table 1:** Patient baseline clinical characteristics

Characteristics	Surgery, no. (%)		*P* value
	Open (*n = *214 %)	Laparoscopy (*n = *216 %)	
Gender			0.411
Men	76 (35.51)	85 (39.35)	
Women	138 (64.49)	131 (60.65)	
Age (years)	59.37 (12.46)	60.40 (10.08)	0.577
Body mass index (kg/m^2^)	23.26 (3.01)	23.46 (3.32)	0.479
Presence of comorbidities			0.425
Yes	70(32.71)	61(28.24)	
No	144(67.29)	155(71.76)	
ASA score			0.336
I	92(42.99)	82(37.96)	
II	119(55.61)	130(60.19)	
III	3(1.40)	4(1.85)	
Clinical tumor (T) category			0.275
T1	17(7.94)	12(5.56)	
T2	56(26.17)	57(26.39)	
T3	77(35.98)	73(33.80)	
T4a	64(29.91)	74(34.26)	
Clinical tumor (N) category			0.813
N0	112(52.34)	108(50.00)	
N1	56(26.17)	58(26.85)	
N2	31(14.49)	37(17.13)	
N3	15(7.01)	13(6.02)	
Clinical TNM stage			0.628
I	53(24.77)	44(20.37)	
II	89(41.59)	90(41.67)	
III	70(32.71)	81(37.50)	
IV	2(0.93)	1(0.46)

**Table 2:** Postoperative pathologic data

Characteristics	Surgery, no. (%)		*P* value
	Open (*n = *214)	Laparoscopy (*n = *216)	
Mean no. of retrieved lymph nodes (SD)	23.8 (13.7)	25.3 (16.5)	0.200
Mean no. of metastatic lymph nodes (SD)	3.33 (7.36)	3.40 (6.96)	0.757
Pathologic tumor(T) category			0.372
T1	59 (27.6%)	51 (23.6%)	
T2	43 (20.1%)	34 (15.7%)	
T3	60 (28.0%)	69 (31.9%)	
T4	52 (24.3%)	62 (28.7%)	
Pathologic tumor(N) category			0.757
N0	97 (45.3%)	91 (42.1%)	
N1	40 (18.7%)	44 (20.4%)	
N2	31 (14.5%)	38 (17.6%)	
N3	46 (21.5%)	43 (19.9%)	
Pathologic tumor(M) category			0.345
M0	211 (98.6%)	209 (96.8%)	
M1	3 (1.4%)	7 (3.2%)	
Pathologic TNM stage			0.786
I	73 (34.1%)	64 (29.6%)	
II	62 (29.0%)	65 (30.1%)	
III	74 (34.6%)	82 (38.0%)	
IV	5 (2.3%)	5 (2.3%)	
Received adjuvant chemotherapy			0.996
Yes	102 (47.7%)	103 (47.7%)	
No	112 (52.3%)	113 (52.3%)	
Chemotherapy regularly			0.607
Yes	50 (23.4%)	46 (21.3%)	
No	164 (76.6%)	170 (78.7%)	

### Oncologic outcomes

**Fig. 2:**
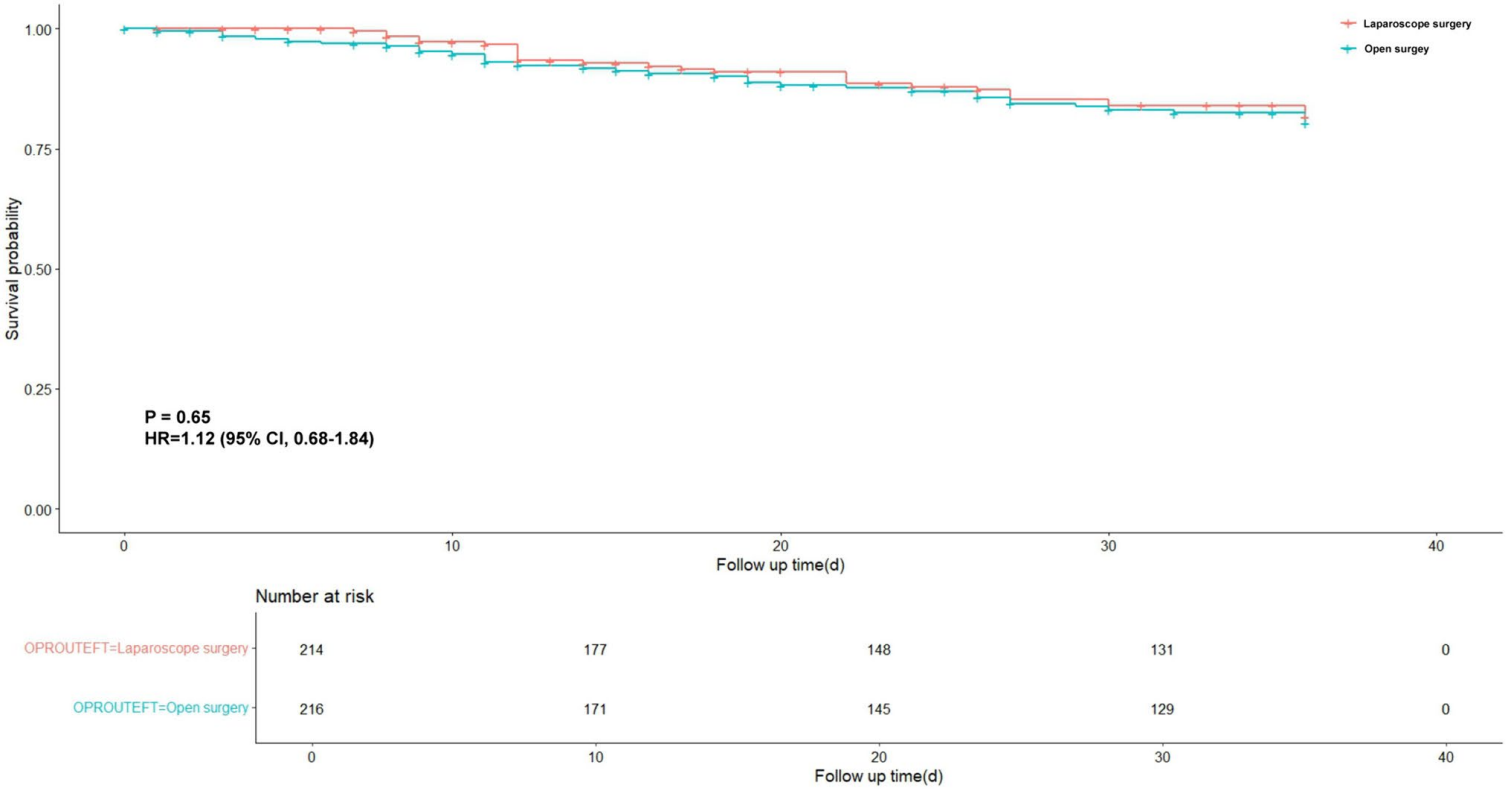
The 3-year DFS rate in the LDG group and ODG group

**Fig. 3:**
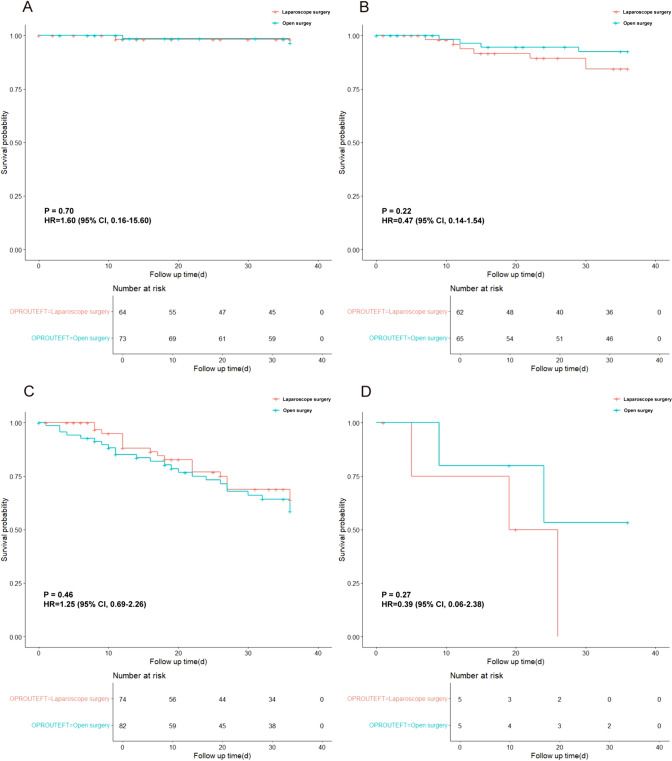
The 3-year DFS rate in the LDG group and ODG group stratified by pathologic staging I (**A**), II (**B**), III (**C**) and IV (**D**)

The 3-year DFS rate in the LDG group was 85.98% and that in the ODG group was 84.72%, with no significant statistical difference between the two groups (Hazard ratio [HR] 1.12; 95% confidence interval [CI] 0.68 – 1.84, *P* = 0.65) (Fig. 2). When stratified by pathologic staging (I–IV), the HR was 1.60, 0.47, 1.25 and 0.39 in each stage without significant difference (*P* = 0.70, 0.22, 0.46, 0.27), respectively (Fig. 3). Similarly, there was no statistically significant difference between the two groups with regards to whether it was pathologic T4aN0 (HR 0.98; 95%CI 0.50–1.92, *P* = 0.95) or T4aN + (HR 1.36; 95%CI 0.16 − 11.30, *P* = 0.95) (Supplement Fig. 1). The number of recurrence was 25 (11.6%) in LDG group, where it was 21 (9.8%) in ODG group; this difference was not statistically significant (*P* = 0.555). Moreover, the sites of recurrence were also similar between the 2 groups with no statistical significance (all *P* > 0.05) (Table 3). Meanwhile, among 48 deathed patients, the 3-year overall survival rate in LDG group was 88.4% (25/216) and that in the ODG group was 90.2% (21/214), with no significant statistical difference (HR 1.26; 95%CI 0.76 − 1.63, *P* = 0.42).

**Table 3:** Frequencies of recurrence and metastasis within 3 years following surgery for patients

Variables	Surgery, no. (%)		*X*^*2*^	*P* value
	Open (*n = *214)	Laparoscopy (*n = *216)		
Any recurrence	21	25	0.349	0.555
Local	2	7	1.778	0.182
Peritoneum	3	5	0.118	0.731
Lung	5	5	0	1.000
Liver	2	1	0	0.994
Multiple sites	8	6	0.315	0.575
Other or uncertain sites	1	1	0	1.000

### Risk factors for survival

Univariate and multivariate analyses of risk factors for disease-free survival have been shown in Table 4. In univariate analyses, BMI < 25 kg/m^2^, pathologic T4 (pT4), pathologic N2-3 (pN2-3), pathologic stage III-IV, and no adjuvant chemotherapy were risk factors for DFS. Furthermore, multivariate analyses demonstrated that BMI < 25 kg/m^2^, pT4, and pN2-3 were the independent risk factors for DFS. Moreover, LDG was not confirmed as the risk factor for DFS when comparing with ODG in this trial.

**Table 4:** Univariate and multivariate Cox regression analyses of risk factors for survival

Characteristics	No. (*n = *430 %)	Disease-free survival			
		Univariate analysis		Multivariate analysis	
		Hazard ratio (95% CI)	*P*	Hazard ratio (95% CI)	*P*
Operative approach		1.03 [0.63,1.69]	0.907		
Laparoscopy	214 (49.77)				
Open	216(50.23)				
Age (years)		1.00 [0.61,1.64]	0.989		
< 60	184 (42.79)				
≥ 60	246 (57.21)				
Gender		0.69 [0.42,1.14]	0.145		
Men	269 (62.56)				
Women	161 (37.44)				
Body mass index (kg/m^2^)		0.04 [0.01,0.27]	**0.001**	0.04 [< 0.01,0.29]	**0.001**
< 25	312(72.56)				
≥ 25	118(27.44)				
Pathologic tumor (T)category					
T1	110(25.58)				
T2	77(17.91)	2.98 [0.75,11.91]	0.123	1.54 [0.36,6.59]	0.563
T3	129(30.00)	5.16 [1.50,17.72]	0.009	2.11 [0.54,8.23]	0.281
T4	114(26.51)	17.27 [5.33,55.99]	** < 0.001**	5.13 [1.34,19.64]	**0.017**
Pathologic tumor (N)category					
N0	188(43.72)				
N1	84(19.53)	1.27 [0.43,3.79]	0.667	0.82 [0.26,2.54]	0.728
N2	69(16.05)	7.17 [3.22,15.97]	** < 0.001**	3.42 [1.41,8.27]	**0.006**
N3	89(20.70)	9.73 [4.62,20.46]	** < 0.001**	4.49 [1.93,10.45]	** < 0.001**
Pathologic TNM stage					
I	137(31.86)				
II	127(29.53)	4.46 [1.24,15.97]	0.022		
III	156(36.28)	17.65 [5.48,56.87]	** < 0.001**		
IV	10(2.33)	37.45 [8.91,157.34]	** < 0.001**		
Received adjuvant chemotherapy		2.51 [1.46,4.30]	**0.001**	1.32 [0.76,2.30]	0.330
Yes	205(47.67)				
No	225(52.33)				

Bold indicates statistical significance

## Discussion

In this multicenter study conducted in northern China, our findings indicate that there were no statistically significant differences in the 3-year DFS between patients with AGC who underwent LDG or ODG with D2 lymphadenectomy. In multivariate analysis, a BMI of less than 25 kg/m^2^, pT4 stage, and pN2-3 status were identified as independent risk factors for patients with AGC following surgery.

Recently, the CLASS group was the first to report that LDG was not inferior to ODG in terms of 5-year OS in treating patients with AGC in China [11]. To our knowledge, this was also the first multicenter RCT to report 5-year survival outcomes for AGC patients globally. However, it is important to acknowledge the significant heterogeneity among gastric cancer patients from North and South China. Patients in the northern region of China are generally more obese than those in the South, due to geographical, dietary, and climatic factors [12]. Based on data obtained from a tertiary hospital in South Korea, it was found that male gastric cancer patients with a higher BMI exhibited significantly better prognoses compared to their female counterparts [13]. Furthermore, Matsui et al. conducted a study indicating that increased visceral fat mass could result in more post-gastrectomy complications; however, it might enhance OS rates for patients with AGC [14]. Notably, there is limited exploration into the disparities in DFS among AGC patients in North and South China.

In the KLASS-01 trials, the oncological outcomes of LDG for early gastric cancer (EGC) were previously presented [15]. The study demonstrates that the 5-year OS rates of LDG and ODG for EGC are similar at 94.2% and 93.3%, respectively, confirming the non-inferiority of LDG for EGC [15]. The advancements in surgical techniques have led to a progressive expansion of laparoscopy’s indications to AGC in eastern countries. Numerous high-quality RCTs have confirmed the safety and feasibility of laparoscopy for treating AGC [3–5]. A recent meta-analysis of five RCTs also demonstrated the equivalence between laparoscopy and open surgery regarding overall short-term morbidity and mortality for AGC [16]. However, unlike laparoscopy for EGC, the oncological prognosis of laparoscopy for AGC remains a concerning and controversial issue. This is primarily due to the complexity of laparoscopic lymph node dissection technology and the biological characteristics of AGC itself. Standardized lymph node dissection plays a crucial role in the oncological prognosis of gastric cancer, particularly in patients with AGC. Although the laparoscopic approach offers enhanced exposure, the role of LDG for AGC remains contentious due to the intricacies of D2 lymphadenectomy during in surgery. However, this study found that neither open nor laparoscopic surgical approaches were identified as risk or protective factors for DFS in cox regression analyses, consistent with the results of the CLASS-01 and KLASS-02 trials.

Disease-free survival (DFS) stands as the predominant endpoint in cancer clinical trials globally. In parallel to the CLASS-01 trials, where a margin of noninferiority of 10% was assumed, the ongoing multicenter investigation in northern China reveals that the 3-year DFS rates for AGC within the LDG group (85.98%) closely mirrors that observed in the ODG group (84.72%). These findings align with recent retrospective and prospective studies examining the oncological outcomes associated with laparoscopic versus open surgery for gastrectomy [17–19]. Meanwhile, using − 8% as the noninferiority margin, the noninferiority of LDG for AGC was also demonstrated by the recent KLASS-02 trials from Korea [9]. Despite the different noninferiority margins used by the clinical trial, their findings implied that LDG with D2 lymph node dissection is an optional treatment strategy for AGC. Furthermore, the subgroup analysis of our trials demonstrated that the rates of 3-year DFS rates on the ODG group and LDG groups for patients with different pathological stages were: stage I- 97.26% and 98.44% respectively; for patients with pathological stage II- 93.85% and 88.71%, respectively; for patients with pathological stage III 69.51% and 74.32%, respectively; and for patients with pathological stage IV 60% and 40%, respectively. In comparison to CLASS-01, this study observed a higher proportion of patients with early stage gastric cancer (31.8% vs. 29.2%) and a lower percentage of patients who underwent adjuvant therapy (39.4% vs. 47.7%). The observed disparity may account for the elevated 3-year DFS rate in this investigation compared to the CLASS-01 cohort, notwithstanding the congruence in inclusion and exclusion criteria between the two studies.

This study diverges from the conclusions drawn in the CLASS-01 and KLASS-02 trials by suggesting that a higher preoperative BMI correlates with improved DFS following distal gastrectomy in patients with AGC. While obesity is commonly identified as a cardiovascular risk factor, its impact on cancer remains equivocal [20].

Numerous studies have investigated the correlation between BMI and the prognosis of patients diagnosed with GC. Specifically, gastric cancer patients with elevated BMIs exhibited more favorable oncological outcomes post-gastrectomy compared to those with normal weight, particularly in Japan and Korea [13, 19]. Elevated BMI was observed to correlate with increased postoperative morbidity [21], however, its role as an independent prognostic factor for patients with AGC in China remains uncertain. Furthermore, previous studies have indicated that a higher BMI does not independently predict long-term survival among patients with GC in Western populations [22]. In our investigation, we have illustrated the adverse oncological prognosis observed in patients with a lower BMI subsequent to AGC diagnosis in northern China. Several potential mechanisms may underlie these findings, including the propensity for tumors to exhibit greater aggressiveness in underweight individuals compared to their obese counterparts. Several studies have demonstrated that individuals with higher BMI exhibit reduced cellular differentiation and decreased occurrence of lymph node metastasis [23, 24]. Also, patients with lower BMI usually have lower muscle mass, which might result in hampered immunity [25]. A meta-analysis conducted by Zhao B et al., which included 12 studies, revealed that underweight preoperative patients with GC were associated with a higher risk of death from causes other than cancer, especially infection [26]. Third, weight loss was often accompanied by gastrectomy or chemotherapy, which could affect DFS. A study reported that ideal body weight can be achieved by overweight patients following curative gastrectomy, which could lead to better long-term prognoses [23]. Therefore, the relationship between BMI and prognosis deserves further attention.

Several limitations of this study warrant consideration. Firstly, the limited participation of hospitals from other provinces in North China may have compromised the generalizability of the findings. Secondly, approximately 10% of patients initially diagnosed with advanced cancer stages were subsequently confirmed postoperatively to have stage I cancer, potentially impacting the reliability of the conclusions. Thirdly, this study lacked measurement of visceral adiposity or muscle mass for assessing obesity, representing a significant limitation. Finally, although there was no significant differences in 3-year DFS rate between the two groups and the outcomes of two groups seem to be comparable at a glance, these results never mean to prove the non-inferiority of LDG to ODG in this study design.

## Conclusions

In conclusion, this study has shown that the 3-year DFS rate among patients with AGC who underwent LDG or ODG with D2 lymphadenectomy in Northern China did not exhibit significant differences.

## Supplementary Information

Below is the link to the electronic supplementary material.

Supplementary file1 The 3-year DFS rate in the LDG group and ODG group with pathologic T4aN0 (A) or T4aN+ (B) (TIF 8106 KB)
