# Supplementary figures and images for: Long-term outcomes of laparoscopic versus open distal gastrectomy for patients with advanced gastric cancer in North China: a multicenter randomized controlled trial
Source: Surg Endosc. 2024 Jul 9;38(9):4976–85. doi: 10.1007/s00464-024-10952-2 (PMC11362494; doi:10.1007/s00464-024-10952-2)

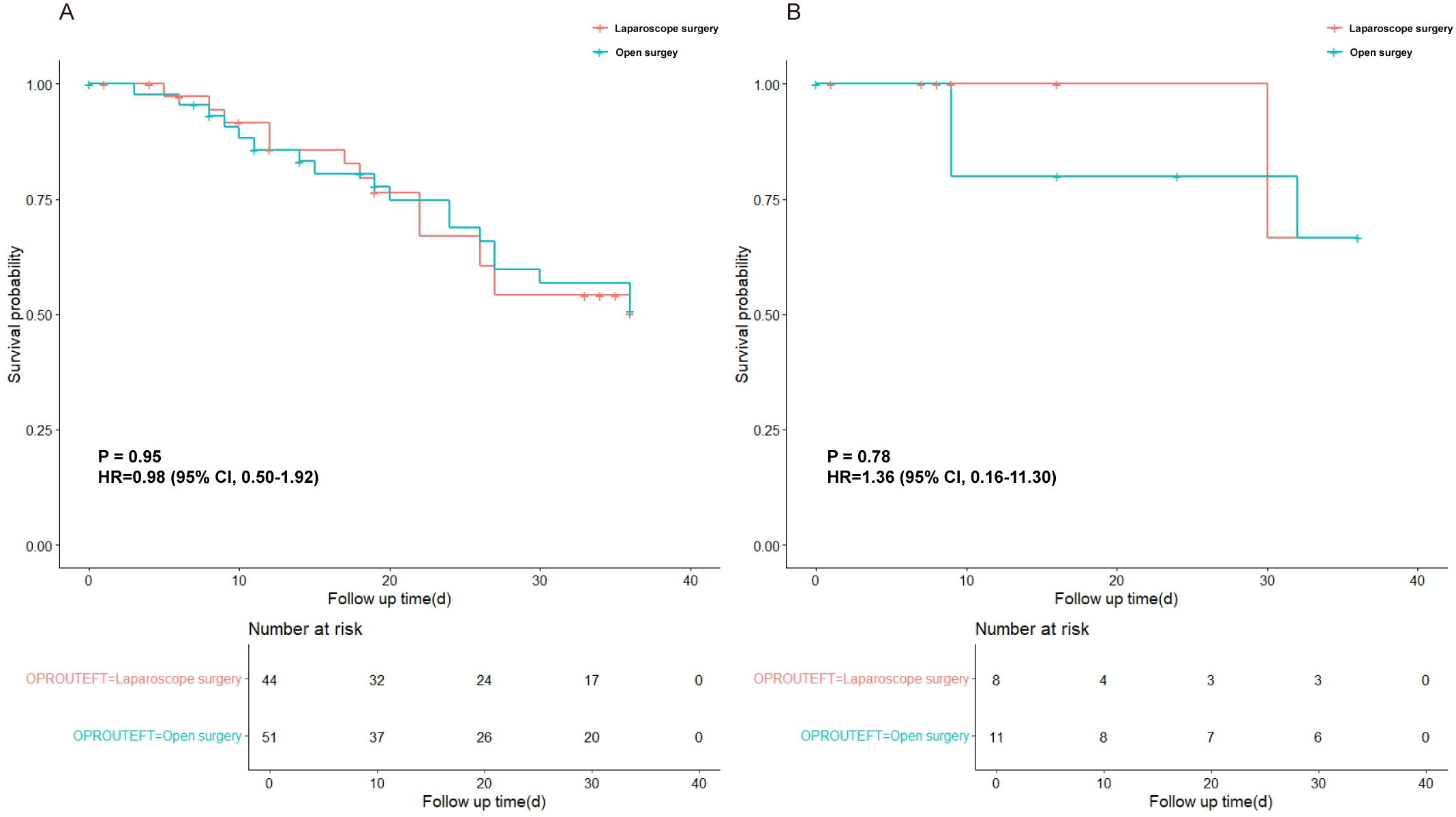

Supplement: Supplementary file 1 — Supplementary file1 The 3-year DFS rate in the LDG group and ODG group with pathologic T4aN0 (A) or T4aN+ (B) (TIF 8106 KB) [file 464_2024_10952_MOESM1_ESM.tif]
